# Overload Control for Signaling Congestion of Machine Type Communications in 3GPP Networks

**DOI:** 10.1371/journal.pone.0167380

**Published:** 2016-12-09

**Authors:** Zhaoming Lu, Qi Pan, Luhan Wang, Xiangming Wen

**Affiliations:** 1 Beijing Key Laboratory of Network System Architecture and Convergence, Beijing, China; 2 Beijing Lab of Advanced Information Network, Beijing, China; 3 Beijing Advanced Innovation Center for Future Internet Technology, Beijing, China; 4 School of Information and Communication Engineering, Beijing University of Posts and Telecommunications, Beijing, China; University of Rijeka, CROATIA

## Abstract

Because of the limited resources on radio access channels of third generation partnership projection (3GPP) network, one of the most challenging tasks posted by 3GPP cellular-based machine type communications (MTC) is congestion due to massive requests for connection to radio access network (RAN). In this paper, an overload control algorithm in 3GPP RAN is proposed, which proactively disperses the simultaneous access attempts in evenly distributed time window. Through periodic reservation strategy, massive access requests of MTC devices are dispersed in time, which reduces the probability of confliction of signaling. By the compensation and prediction mechanism, each device can communicate with MTC server with dynamic load of air interface. Numerical results prove that proposed method makes MTC applications friendly to 3GPP cellular network.

## Introduction

With rapid development of information and communication technologies, machine type communication (MTC), which means that communication is not only required to transmit information between human to human (H2H) but also machine to machine (M2M), has been regarded as emerging technology to change our living styles. MTC servers will connect a huge number of MTC devices through the Internet to form various MTC applications [1][2]. Third Generation Partnership Projection (3GPP) network is always taken as the reliable large-scale deployment solution of machine type communication for its ubiquitous property. Compared with other short distance communication methods, 3GPP infrastructure is mature, ubiquitous, carrier class and easy to deployment, which is reliable and economical for machine type communication.

However, it does not imply a successful practice of MTC devices in 3GPP cellular networks as they are originally designed for voice call and data transmission for Human-to-Human. Instead, there comes a number of challenges. One major challenge lies in the radio access network (RAN). The number of MTC devices may be enormous while the radio resources on RAN are constrained [3]. Therefore, signaling congestion may happen due to simultaneous attempts from a potential number of MTC devices in one cell to attach or connect to the network all at once. One of the typical scenarios is the smart meter devices in buildings which keep idle state in most of time for energy saving and wake up periodically to transmit or receive a determined amount of data at a settled frequency (e.g., every day at 24:00h).

Overload control for signaling congestion in cellular RAN network has recently attracted great attention in MTC research and standardization. Considering that the radio access network is essential for M2M communications, 3GPP organized a work item to trigger the standardization progress for RAN of MTC [1][2]. There are two main possible solutions: one devotes to adapt 3GPP RAN protocols to MTC applications from the perspective of network operators, the other expects to reduce the probability for large number of MTC devices of accessing the network successfully and make MTC applications friendly from the perspective of service providers.

Congestion avoidance is the most common solution for congestion control from the perspective of network operators. Several traditional solutions for congestion avoidance have been discussed, such as backoff based scheme [4], access class barring (ACB) scheme [5][6][7], separating RACH (Random Access Channel) resources [8], dynamic allocation of RACH resources [9]. However, backoff based scheme and separating RACH resources provide performance improvement are only under a light congestion level. The ACB based scheme may lead to unacceptable response time delay, and the performance of dynamic allocation of RACH resources scheme is limited by the availability of additional resources. In paper [10], packet scheduling based on the time-controlled M2M feature in Long Term Evolution (LTE) MAC layer is used to guarantee Quality of Services (QoS) requirements of M2M applications. Unfortunately, these schemes expect to deal with M2M communication with mechanism as same as that of H2H communications. Energy consumption caused by waiting for transmission (MTC devices need to wake up and wait for transmitting opportunity), should be considerable, when massive MTC devices request to communicate to MTC servers at the same time. That is, these schemes may lead to amount of additional energy consumption of MTC devices, which will decrease the lifetime of these battery powered devices. Hence, we try to propose an effective access mechanism for MTC devices to communicate with remote MTC servers, which is friendly to 3GPP RAN, to solve the signaling congestion problem caused by massive synchronous communications with minimized energy consumption, and guarantee the QoS of MTC applications. In brief, solutions from the point of 3GPP radio network are restricted by the constrained resources in RAN. Attempting to solve this problem through one certain scheme is unpractical. Unfortunately, few literatures have investigated in the scheme to make MTC applications friendly for cellular RAN. As a result, we propose a signaling overload control with periodic reservation (SOC-PR) algorithm for various MTC applications in the point of service providers.

There are three contributions in this paper. Firstly, through trend fitting model based statistical learning of MTC server, a 3GPP network friendly signaling congestion avoidance mechanism is proposed to periodically predict the available access resources for MTC applications. Secondly, MTC applications proactively reserve authorized time windows for the MTC devices, and periodically disperse the incoming connect requests to ensure an even distribution. The confliction on RAN due to simultaneous requests from massive MTC devices in one cell is highly reduced. Meanwhile, the energy consumption caused by retransmission of each MTC device for conflictions is reduced. Thirdly, QoS of MTC applications is guaranteed by this network friendly mechanism.

## System Model

The lack of an unfailing supply of energy is a main challenge restricting most of the MTC applications. In H2H communications, battery can easily be charged in the phone. However, it is quite another thing in machine to machine communication since MTC devices are always deployed to places not easily reached as well as the large amount of the MTC devices. In addition, MTC devices shall be working for a very long time (may be 4–5 years). Therefore, MTC devices need effective energy efficient mechanism to extend the battery life. Hence, MTC devices keep idle state in most of time and wake up periodically to report messages to MTC server. By this mechanism, energy efficient operation can be achieved. In the context of MTC, signaling confliction may happen due to this periodically activation of massive devices. Synchronous access requests of large number of MTC devices in one cell may lead to congestion of cellular RAN [11]. Therefore, the signaling congestion problem should be solved in an energy efficient way, as well as assuring the QoS of MTC applications. In this paper, our focus is mainly on the case in which MTC devices establish the connection with the remote MTC server via the 3GPP cellular network, such as LTE/LTE-A.

**Fig 1:**
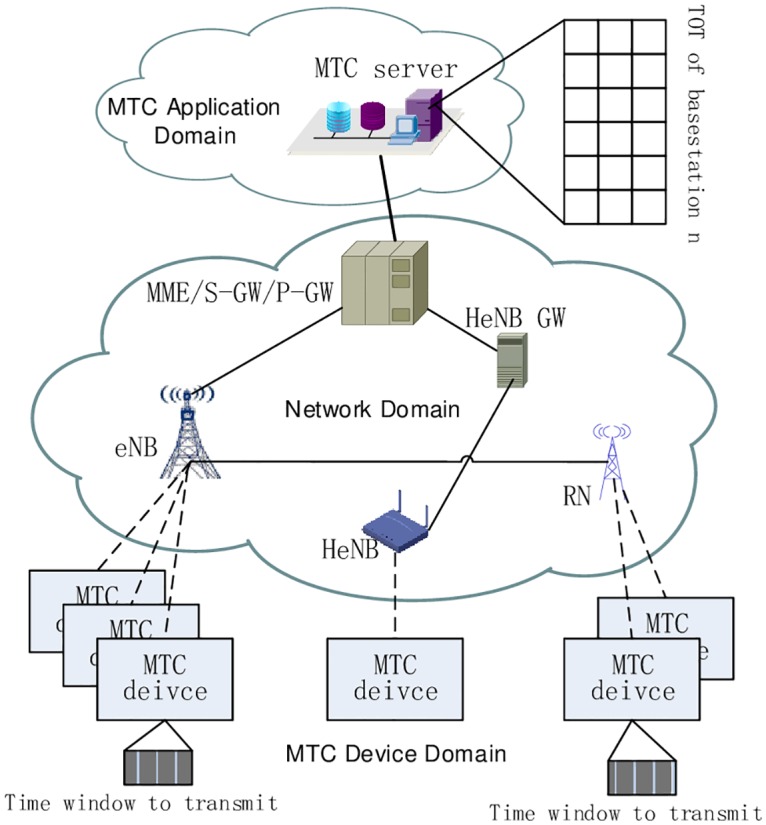
Scenario for machine type communication.

As depicted in Fig 1, this paper mainly studies the MTC devices to MTC server communication scenario [1] which represents the most envisaged one. It consists of three main domains, including the MTC device domain, the network domain and the MTC application domain. Depending on the use case, the MTC devices transmit or receive a determined amount of data at a determined frequency or period. For example, a smart meter device sends measurement results every day at 12pm. MTC devices are always fixedly installed in certain position or with limited mobility. The communication network domain can be a wired or a wireless network. In this paper, our focus is on the case where the communication network is a 3GPP mobile network, such as LTE/LTE-A. As shown in Fig 1, the base stations (BSs) communicating with MTC devices can be eNodeB (eNB), Home eNodeB (HeNB), Relay Node (RN) and so on. Suppose there are *N* (indexed by *i* = 1,⋯, *n*, ⋯, *N*) base stations correlated with the MTC application we study. The MTC application domain consists of MTC servers, under the control of the mobile network operator or a third party. Applications with certain time tolerance are investigated in this paper. The MTC server can be aware of the delay sensitivity, communication mode between devices and server, charging method of its applications. According to these characteristics, proper overload control strategy is needed to be adopted for signaling congestion in 3GPP cellular networks.

## Overload Control Method Based on Periodic Reservation

To reduce the energy consumption during the MTC signaling communications, an energy efficient model constrained by QoS is proposed in this paper, and a periodic resource reservation method is adopted to achieve the sub-optimal solution of this model.

### A. Energy efficient model constrained by QoS

The MTC applications mentioned in this paper is mainly about the communications in which MTC devices always access the network periodically. With regard to one base station *BS*_*n*_ providing radio access link for this application, suppose there are *M* (indexed by 1,⋯, *m*, ⋯, *M*) MTC devices served by the base station *BS*_*n*_ in this MTC application. One time window *TW* represents the time interval in which MTC device can be allowed to communicate with MTC server. When the air interface is totally occupied in one time window, new access request of MTC devices will be rejected. The rejected devices need to stay waking up to wait for opportunity for transmission, which will lead to additional energy consumption of MTC devices.

A network friendly periodic reservation scheme should be proposed to reduce the energy consumption caused by signaling congestion, and guarantee the QoS of MTC applications.

$$\{\begin{array}{c} \text{min}\sum p_{m},m=1,\cdots ,M\\ tw_{last}^{\tau }-tw_{first}^{\tau }<Th_{delay},\tau =1,\cdots T\\ num_{device}^{tw}\underset{\text{max}}{\propto }ch_{access}^{tw} \end{array}$$ (1)

Where *p*_*m*_ denotes the energy consumption of MTC device *m*. Energy consumption of one certain MTC device is dependent on its awake time length. $tw_{last}^{\tau }$ and $tw_{first}^{\tau }$ denote the last time window for device transmitting data and the first time window for device transmitting data respectively in period τ. *Th*_*delay*_ is depicted the QoS requirement for specific MTC application. $num_{device}^{tw}$ and $ch_{access}^{tw}$ denote the number of synchronous communication devices in one time window and the number of available access channels in one time window respectively. $\underset{\text{max}}{\propto }$ is expressed the maximum consistency. Obviously, these three formulas denote the energy efficiency, QoS requirements and network friendliness of proposed model. The proposed mechanism tries to minimize the energy consumption and time delay through periodic transmitting opportunity reservation. According to these objectives to optimized, the proposed mechanism is designed as the following section.

### B. Periodic resource reservation method

As the resources in 3GPP RAN are limited, this overload control scheme must be friendly to 3GPP RAN, which is implemented through periodic statistical learning. In signaling overload control mechanism with periodic reservation (SOC-PR), the MTC server maintains a transmission occupancy table (TOT), which records the number of MTC devices assigned to wake up at each time window, for each served base station. That is there are *N* tables for this application. For base station *BS*_*n*_, the corresponding TOT is *T*_*n*_. As the duration of transmission for MTC devices is always very short, the traffic load of non-MTC could be supposed to be stable. Because the MTC devices of applications investigated in this paper are fixedly installed in certain position. The length of the *nth* TOT (number of time windows in the table) of period τ,$L_{Tn}^{\tau }$, is an adaptive parameter based on statistical learning of MTC server. As the variation of TOT length is in line with traffic load of radio access networks, which has obvious trends with time for carrier grade networks. MTC server adopts the trend fitting model to predict $L_{Tn}^{\tau }$ periodically by

$$\{\begin{array}{c} L_{Tn}^{\tau }=\left\lceil \alpha +\beta \tau +I_{\tau }\right\rceil ,\tau =1,2,\cdots ,S\\ E\left(I_{\tau }\right)=0,Var\left(I_{\tau }\right)=\sigma^{2} \end{array}$$ (2)

Where *S* denotes the number of sample of TOT length. *α* and *β* are parameters for trend fitting model, which are estimated by least square method. ⌈•⌉ denotes rounding up to an integer. As the length of the *nth* TOT is predicted by trend fitting model, which may not be always optimal solution in each prediction, so that the SOC-PR method can just achieve the sub-optimal solution of energy efficient model constrained by QoS.

Therefore, consider a MTC device *MTC*_*m*_, before *MTC*_*m*_ enters idle state for power saving, it transmits a time assignment request to the MTC server. On receiving a time assignment request, the MTC server use its current timestamp, *TS*, to calculate the current position in TOT, $tw_{l}^{\tau }$, in the TOT by

$$tw_{l}^{\tau }=\left\lceil \frac{TS}{TWlength}\right\rceil \text{mod}L_{Tn}^{\tau }$$ (3)

Where *TWlength* is the length of each time window in *ms*.

The base station *BS*_*n*_ searches the TOT from the beginning of the table forward to find an entry, $tw_{l}^{\tau +1}$, with the number of MTC devices assigned less than predicted available access number $I_{lt}^{\tau }$. It calculated by

$$I_{lt}^{\tau }=\left\lceil M/L_{Tn}^{\tau }\right\rceil$$ (4)

MTC server then calculates the offset of this MTC device, *offset*_*m*_, by

$$offset_{m}=\left(\left(L_{Tn}^{\tau }+tw_{l}^{\tau +1}-tw_{l}^{\tau }\right)\text{mod}L_{Tn}^{\tau }\right)$$ (5)

The base station *BS*_*n*_ replies to MTC device with a time assignment confirm which carries the offset value *offset*_*m*_ of this device. The corresponding entry of the TOT, $BOT(tw_{l}^{\tau +1})$ is increased by 1.

When MTC device receives the time assignment confirm, it changes into idle state, and wake up after *offset*_*m*_ to communicate to MTC server. From then on, *MTC*_*m*_ will follow its schedule and wake up periodically to update the position in TOT and communicate to the MTC server.

Besides the periodic reservation strategy of overload control for signaling congestion, a compensation mechanism is also adopted to cooperate with it. In each period, if there are not enough access channels for MTC device *MTC*_*m*_, this device will be rejected in its reserved time window by served base station. It will take a backoff to solve the conflict. The backoff length is set as the length of TOT.

$$backoff_{m}^{\tau }=L_{Tn}^{\tau }$$ (6)

By this method, as each access request of MTC device is arranged reserved resource, there will be less probability of conflict. Every MTC device could wake up at the right time. Delay for each request will decrease and the energy consumption of MTC devices will decrease, too.

## Numerical Results and Discussion

In this section, we evaluate the performance for SOC-PR based on parameters of LTE-Advanced radio access networks. The traffic model is assumed to be mobile originated, meaning that the MTC server will not poll/request reports from the MTC devices. T1 [12] traffic model is adopted in this paper. The path loss model is a typical distance dependent model. Network and MTC devices parameter settings in this simulation are referred to the typical parameter configurations depicted in table 1 [13][14]. Each MTC device occupies 5 RBs (resource blocks) to transmit data to MTC server with packet size 1.28kb and 16QAM is adopted as the modulation scheme. We assume there are 500, 1000 and 3000 MTC devices served by one base station, imitating the massive and different quantity of access attempts for 3GPP network.

**Table 1:** Parameters for evaluations.

Parameter	Setting
Number of MTC devices of one base station	500; 1000; 3000
Subframe length	1ms
Cell bandwidth	5MHz
RB number for each MTC device	5
Length of time window	5ms
Evaluation times	1000

In order to indicate the performance of proposed signaling congestion avoidance mechanism SOC-PR, traditional randomized access dispersion scheme [7][15] with fixed resource for MTC applications, which didn’t adopt the periodic resource reservation strategy, is considered in this section. We simulate the traditional randomized access dispersion scheme with different number of time windows for access dispersion. In addition, every final simulation results are the average results got from 1000 times’ evaluation for sure of the accuracy and reliability of this simulation.

To evaluate the performance of SOC-PR, we define two metrics as follows:

**Probability of Confliction**, defined as the probability for MTC devices conflicting with each other when requesting accesses to remote server, which denotes the probability MTC devices successfully complete the radio access procedure. Probability of Confliction (PoC) can be calculated by

$$PoC=N_{conflict}/N_{total}$$

Where *N*_*conflict*_ denotes the number of MTC devices which suffered the confliction in each periodic time window, while *N*_*total*_ denotes the total number of access attempts for network.

**Fig 2:**
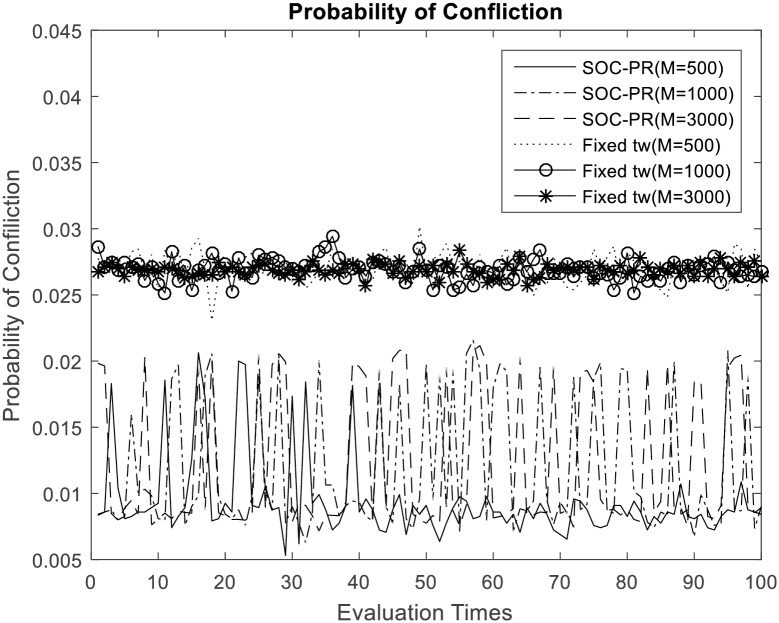
Probability of confliction with different number of devices.

**Fig 3:**
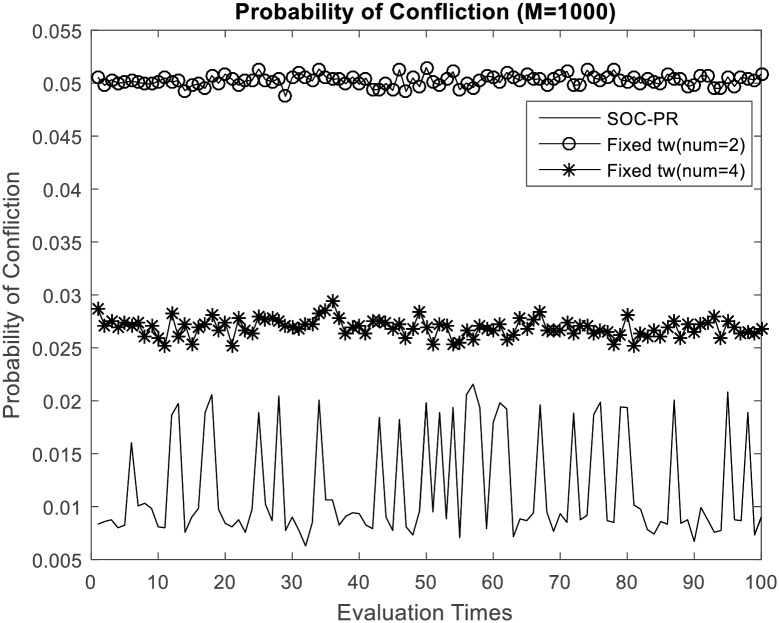
Probability of confliction comparison with the traditional access dispersion mechanism (M = 1000).

As depicted in Fig 2, because massive accesses are spread onto a long period of time for MTC applications, average probability of confliction will stay stationary with the number increasing of MTC device severed by one base station. We can observe that the probability of confliction is no more than 0.02 even if the number of MTC devices is particularly large. Fig 2 also show that proposed SOC-PR scheme performs less probability to conflict than traditional access dispersion scheme with fixed number of time windows when applying to the same number of access attempts. It is apparent that the SOC-PR scheme is friendly to 3GPP network, which does not only disperse access requests in time domain, but also predict the non-MTC traffic periodically through statistical learning. Just as shown in Fig 3, the comparison between the proposed access scheme and traditional mechanisms with different fixed time windows is really evident when the number of access attempts from MTC devices is 1000.

The probability of confliction for each MTC devices keep a low level all the time with the periodic reservation strategy which can adaptively adjust number of the time windows according to the dynamic load of air interface. That is, the consistency between 3GPP RAN and signaling congestion avoidance mechanism is achieved. Further, assume that the MTC devices work in fixed power level when they wake up to transmit data, so longer time duration will lead to more energy consumption. As this network friendly mechanism reduces the probability of conflict of MTC devices, additional backoff time could be decreased as well. Energy consumption caused by confliction is reduced, and the energy consumption of each MTC device could achieve a suboptimal value. In addition, this signaling avoidance scheme is very simple, which has little calculation load on the MTC devices. Hence, our overload control strategy is an energy efficient scheme as well. Therefore, SOC-PR brings less energy consumption for MTC devices, and the life time of them will be highly prolonged.

**Access Delay,** defined as the average time delay that all MTC devices accomplish communication with MTC server in one period, which denotes the average time delay caused by dispersing of access requests. Access Delay (AD) can be calculated by

$$AD=\frac{\sum delay_{m}}{M}$$

Where *delay*_*m*_ denotes the time delay of MTC device *m*.

**Fig 4:**
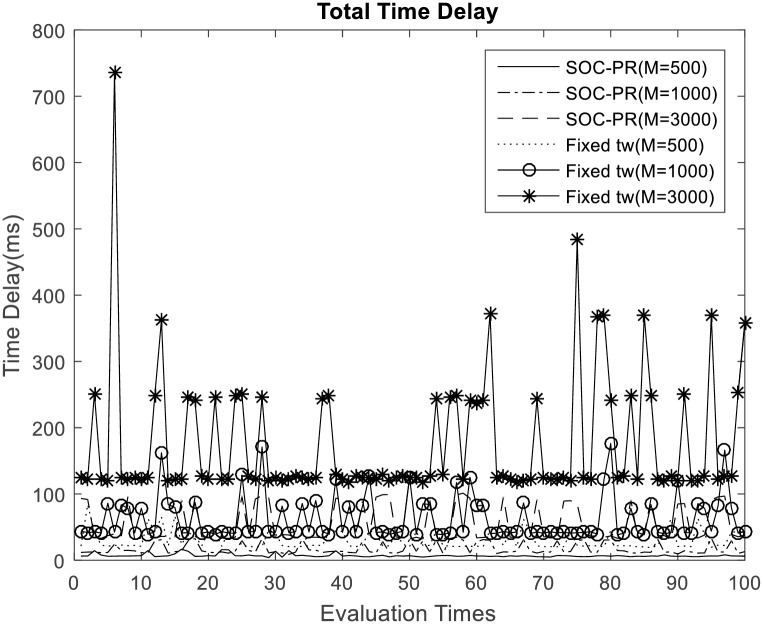
Access delay with different number of MTC device.

**Fig 5:**
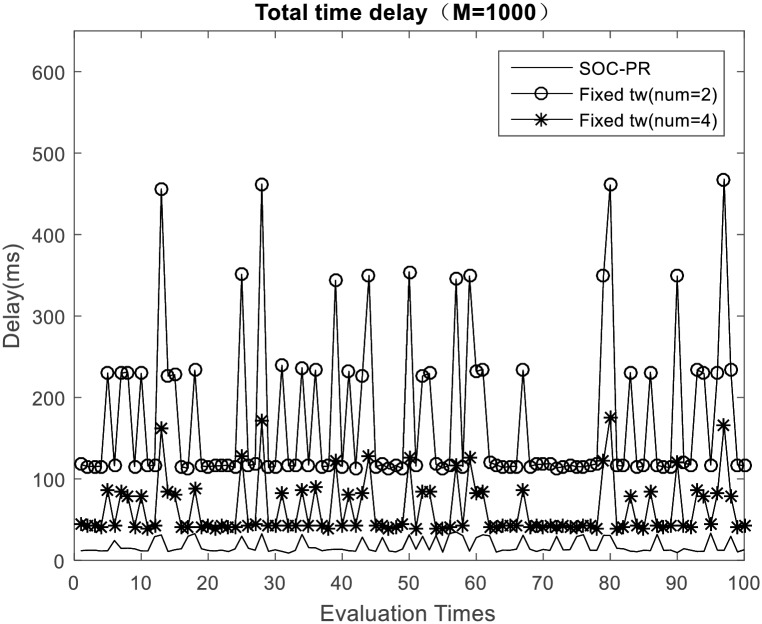
Access delay comparison with the traditional access dispersion mechanism (M = 1000).

As depicted in Fig 4, if number of MTC devices increases, the average time delay resulted by dispersing of access requests will become bigger. When the number of MTC devices be large (e.g. 3000 MTC devices of one application), the backoff time for each access confliction, which is due to the length of TOT in proposed SOC-PR scheme (or the number of devices in traditional dispersion scheme), will become large. Hence, the average delay time will increase with number of devices, and the variance of average delay time will also increase. In Fig 5 when the number of access requests reaches 1000, it can be shown that proposed SOC-PR scheme performs less access delay than traditional access dispersion scheme with the same number of MTC devices, which can also be obtained from Fig 4. Note that even if the number is particularly large the average time delay is still no more than 90ms with the proposed mechanism. However, average time delay in the traditional scheme almost reaches up to 500ms when the fixed number of time windows for dispersion is 2. It is quite obvious that the proposed overload control mechanism for signaling congestion of machine type communications based on SOC-PR is friendly to 3GPP networks, and it can effectively guarantee the QoS of MTC applications.

## Conclusion

In this paper, we solve the most critical issue on the radio access network of 3GPP for machine type communication with the proposed SOC-PR access mechanism. Through periodic reservation strategy, massive access requests of MTC devices are dispersed in a relatively long time period, which reduces the probability of confliction of signaling transmission. By the compensation and prediction mechanism, each device can communicate with MTC server with dynamic load of air interface. What is more, the energy consumption of MTC devices can be minimized with the reduction of the number of waiting time for transmission and retransmission. Simulation results demonstrate that our proposal provided apparent mitigation on the congestion in RAN with higher QoS guarantees for common MTC applications.

## Supporting Information

S1 File (PDF)
