# Supplementary material for: Overload Control for Signaling Congestion of Machine Type Communications in 3GPP Networks
Source: PLoS One. 2016 Dec 9;11(12):e0167380. doi: 10.1371/journal.pone.0167380 (PMC5147883; doi:10.1371/journal.pone.0167380)
Supplement: S1 File — (PDF) [file pone.0167380.s001.pdf]

PLOS

Figure file quality report: 2016-08-15				
Original Filename	PACE Filename	Status	Error Detail(s)	PACE Adjustments
Figure1-1		✓	• No Error	• DOC file is converted to a TIF for submission. Please inspect the tif version for image clarity and content.
Figure2-1		✓	• No Error	• DOC file is converted to a TIF for submission. Please inspect the tif version for image clarity and content.
Figure3-1		✓	• No Error	• DOC file is converted to a TIF for submission. Please inspect the tif version for image clarity and content.
Figure4-1		✓	• No Error	• DOC file is converted to a TIF for submission. Please inspect the tif version for image clarity and content.
Figure5-1		✓	• No Error	• DOC file is converted to a TIF for submission. Please inspect the tif version for image clarity and content.
